# Structure of Chorismate Mutase-like Domain of DAHPS from *Bacillus subtilis* Complexed with Novel Inhibitor Reveals Conformational Plasticity of Active Site

**DOI:** 10.1038/s41598-017-06578-1

**Published:** 2017-07-25

**Authors:** Shivendra Pratap, Aditya Dev, Vijay Kumar, Ravi Yadav, Manju Narwal, Shailly Tomar, Pravindra Kumar

**Affiliations:** Department of Biotechnology, Indian Institute of Technology Roorkee, Roorkee, 247667 India

## Abstract

3-deoxy-D-arabino-heptulosonate-7-phosphate-synthase (DAHPS) is the first enzyme of the shikimate pathway and is responsible for the synthesis of aromatic amino acids in microorganisms. This pathway is an attractive target for antimicrobial drugs. In *Bacillus subtilis*, the N-terminal domain of the bifunctional DAHPS enzyme belongs to an AroQ class of chorismate mutase and is functionally homologous to the downstream AroH class chorismate mutase. This is the first structure of chorismate mutase, AroQ (*Bs*CM_2) enzyme from *Bacillus subtilis* in complex with citrate and chlorogenic acid at 1.9 Å and 1.8 Å resolution, respectively. This work provides the structural basis of ligand binding into the active site of AroQ class of chorismate mutase, while accompanied by the conformational flexibility of active site loop. Molecular dynamics results showed that helix H2′ undergoes uncoiling at the first turn and increases the mobility of loop L1′. The side chains of Arg45, Phe46, Arg52 and Lys76 undergo conformational changes, which may play an important role in DAHPS regulation by the formation of the domain-domain interface. Additionally, binding studies showed that the chlorogenic acid binds to *Bs*CM_2 with a higher affinity than chorismate. These biochemical and structural findings could lead to the development of novel antimicrobial drugs.

## Introduction

Chorismic acid, the precursor for a wide range of aromatic compounds, including aromatic amino acids, folate, and ubiquinones, is essential for the survival of bacteria and is synthesized through the shikimate pathway^1^. The shikimate pathway is present in microorganisms, plants, and apicomplexan parasites, but is absent in higher eukaryotes. Altogether, these properties make enzymes of this pathway promising targets for the development of antibiotics, herbicides and pesticides^2, 3^. The first step of the pathway, aldol condensation of phosphoenolpyruvate (PEP) and D-erythrose-4-phosphate (E4P) to 3-deoxy-D-arabino-heptulosonate-7-phosphate (DAHP) is catalyzed by the DAHP synthase enzyme^4^. DAHP synthase activity is regulated by feedback inhibition to control the substrate input with respect to the cellular concentration of downstream reaction products of the pathway^4–6^. Different organisms have evolved to regulate DAHP synthase activity through different allosteric mechanisms and associated allosteric machinery^7, 8^.

All structurally characterized DAHPS enzymes share a characteristic (β/α)_8_ TIM – barrel fold and similar active site architecture^9–12^. Initially, based on variations in amino acid sequences, DAHPS enzymes were classified into different classes, including type Iα, type Iβ and type II. With the availability of structural information, these sequence variations were attributed to different structural additions to the core catalytic barrel^9, 13–16^. In *Escherichia coli*, three isoenzymes of DAHPS, type Iα enzymes, are present, and each isoenzyme is responsive to phenylalanine, tyrosine or tryptophan^17^. *Mycobacterium tuberculosis* DAHPS, a type II enzyme, has a synergistic mode of inhibition caused by binding of tyrosine and tryptophan or phenylalanine and tyrosine at discrete allosteric sites^6^. Additionally, noncovalent complex formation between chorismate mutase (CM) and DAHPS in *M. tuberculosis* modifies allosteric regulatory properties of both the enzymes through molecular symbiosis^15, 18^. Chorismate, a substrate of CM, inhibits DAHPS activity, which suggests that CM has a regulatory role in this complex^19^. In *Thermotoga maritima*, DAHPS, a type Iβ enzyme, activity is regulated by an N-terminal ferredoxin-like (FL) regulatory domain. Aromatic amino acids, phenylalanine or tyrosine binds to the FL domain and cause a conformational change which restricts substrate access to the active site^5, 20, 21^.

A DAHPS enzyme from *Listeria monocytogenes* has an N-terminal CM, type II domain (*Lm*CM), which regulates the enzyme’s activity through binding of chorismate or prephenate^9^. The regulatory *Lm*CM domain is entirely helical in structure and is classified as a “Chorismate Mutase II fold” in the SCOP database^22^. The regulatory domain is connected to the DAHPS domain through a linker region. Light *et al*. have also summarized the possible mode of allosteric regulation, such as (i) domain linker mediated transmission of inhibitory conformational changes or (ii) domain-domain inhibitory interaction stabilization^9^. Domain-domain interface interactions are mediated by polar and hydrophobic contacts between catalytic and regulatory domains. In *L. monocytogenes*, residues Phe46, Leu49, Arg52, and Lys76 of the regulatory domain participate in domain-domain interface interactions with Phe163, Asp326, and Val329 of the catalytic domain^9^. The N-terminal domain of bifunctional P-protein from *E. coli* (*Ec*CM) also has a chorismate mutase II fold^23, 24^. Although, *Ec*CM shares same fold as *Lm*CM, it is a fully functional enzyme, unlike *Lm*CM.

A recently published crystal structure of DAH7PS from *Geobacillus sp*. shares a similar architecture, with an N-terminal CM domain, which is shown to be catalytically active^25^. The full-length protein exhibits the activity of both enzymes, but interestingly, in the absence of the CM domain, the DAHPS activity is significantly increased, whereas the separated CM domain is catalytically less active^25^. This observation clearly implies that the CM domains interacts with the DAHPS domain and possess a regulatory role in the wild-type enzyme. Furthermore, Nazmi *et al*. showed the inhibition of DAHPS domain activity in the wild-type enzyme in the presence of prephenate, the product of CM^25^. This inhibitory activity was not observed for a separated DAHPS domain. Based on the prephenate bound DAHPS crystal structure, in addition to SAXS analysis, Nazmi *et al*. proposed that in the unbound state, the CM domain adopts a open conformation and allows the DAHPS substrate to access its active site. In contrast, binding of prephenate to the CM domain induces conformational changes resulting in a more compact closed conformation, which bring both domains close to each other. This structural rearrangement results in obstruction of the DAHPS active site thereby inhibits its activity.

The N-terminal domain of DAHPS from *B. subtilis* is homologous to the AroQ class of chorismate mutase, type II^26, 27^. However, *B. subtilis* also contains a monofunctional AroH class of chorismate mutase situated downstream of the shikimate pathway^28^. Chorismate mutase converts chorismic acid into prephenate, which is further converted into the aromatic amino acids tyrosine and phenylalanine. The N-terminal domain of DAHPS has minimal catalytic activity, and CM’s substrate/product have been shown to inhibit the activity of the *Bs*DAHPS enzyme^29, 30^. Previously, it was argued that the N-terminal domain has a regulatory function rather than a catalytic one^16, 26, 27^. Chlorogenic acid (CGA), one of the most abundant polyphenols in human diet, is the ester of caffeic acid and quinic acid. CGA decreases the incidence of chemical carcinogenesis in animal models, and also inhibits bacterial growth^31, 32^. However, the molecular mechanism of its anticancer and antibacterial activity is largely elusive.

In the present study, based on our biochemical and structural findings, we show that chlorogenic acid, a structural analogue of chorismic acid, is an inhibitor of chorismate mutase, type II regulatory domain (*Bs*CM_2) of DAHPS from *B. subtilis*. Crystal structures of *Bs*CM_2 in complex with citrate and chlorogenic acid were determined at 1.9 Å and 1.8 Å, respectively. The present study is the first report of the AroQ class of *Bs*CM_2 structures and provides insight into active site architecture and its regulatory role. Molecular dynamics simulations were performed to observe active site loop flexibility. Through structural analysis, we have provided explanations of the minimal catalytic activity of *Bs*CM_2 and other previously reported observations regarding its role in DAHPS activity regulation. This data also provides evidence to reinforce the domain-domain interface hypothesis of DAHPS regulation. Additionally, we also determined kinetic parameters of inhibitory activity of chlorogenic acid and its minimum inhibitory concentration against *B. subtilis*.

## Results

### Overall structure

*Bs*CM_2 crystals complexed with citrate (*Bs*CM_2-CIT) and substrate analogue chlorogenic acid (*Bs*CM_2-CGA) diffracted up to 1.9 Å and 1.8 Å resolution, respectively. *Bs*CM_2-CIT and *Bs*CM_2-CGA structures contain four and two monomers per asymmetric unit, respectively. The *Bs*CM_2 protein has an all-helix entirely helical tertiary structure. In the *Bs*CM_2-CGA structure, the electron density of first N-terminal residue was missing from both the chains. Additionally, residues 2,3 and 40 could not be modeled due to the absence of any interpretable electron density in chain A. The *Bs*CM_2-CIT structure also has some missing residues (residue 1 from chain C; 87 from chain A; and 88 from chain A and D), which were not modeled. The following analysis was performed using *Bs*CM_2-CGA structure unless specified otherwise.

**Figure 1:**
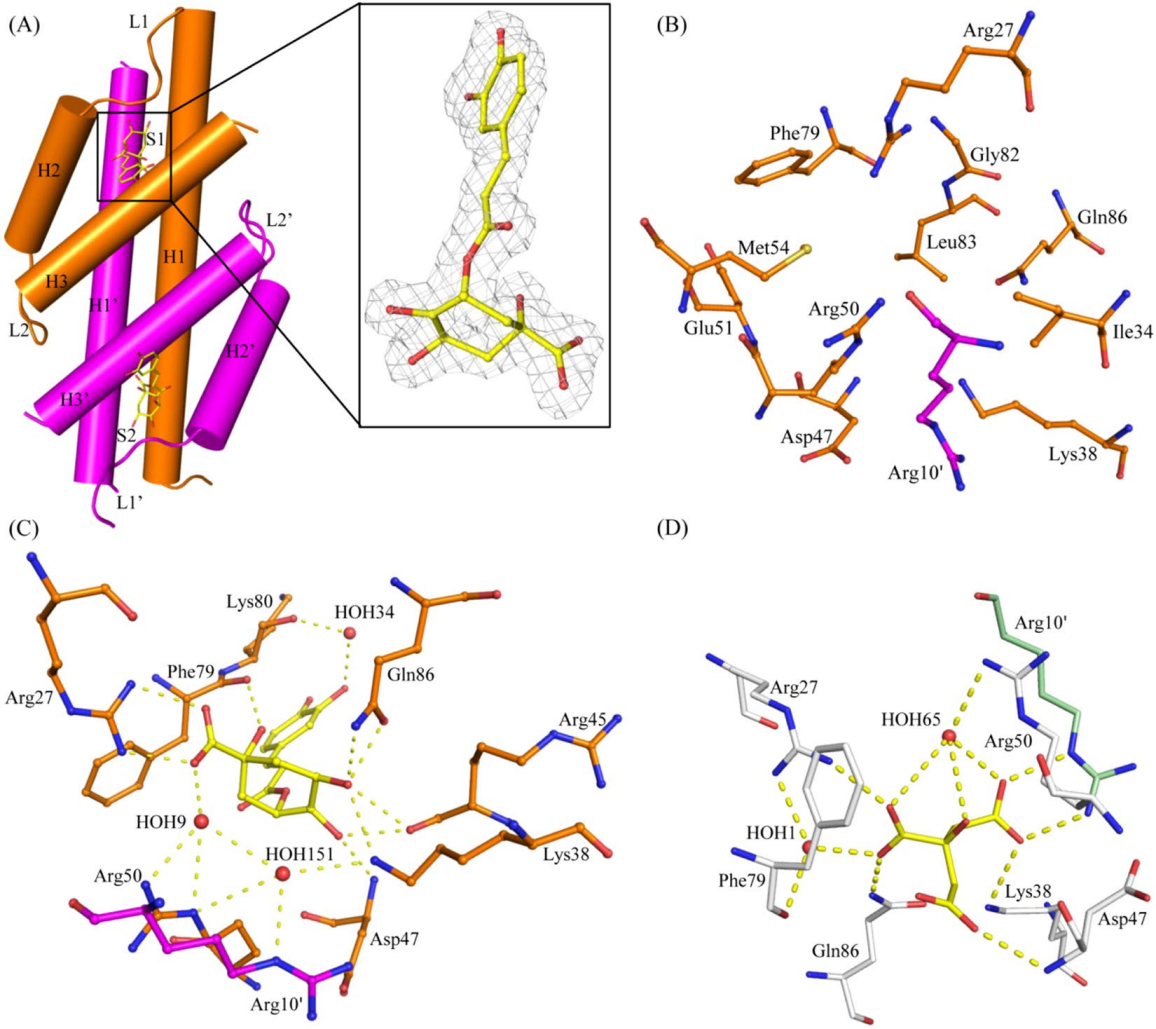
Overall structure, active site and ligand interactions. Panel (A) shows the overall structure of *Bs*CM_2 complexed with chlorogenic acid. In the zoom panel, 2F_0_-F_c_ map of chlorogenic acid at active site S1 contoured at one sigma. In panel (B), the active site is formed by residues contributing from both the chains of physiological dimer. The polar residues at the active site are highly conserved in all CM_2 or CM-like domains. However, hydrophobic acid residues are not conserved. Panel (C) shows the interaction of chlorogenic acid with the active site residues in *Bs*CM_2-CGA structure. Panel (D) shows the interaction of citrate with the active site residues in the *Bs*CM_2-CIT structure. Residues from different chains are colored accordingly.

In *Bs*CM_2–CGA, the monomeric structure contains a ‘Chorismate Mutase, type II fold’, a three-helix bundle connected through the loops. In chain B, three helices H1, H2, and H3 are composed of residues 5–40, 48–60 and 68–87, respectively. Helix H2 is connected to the helices H1 and H3 by 7 residues long loops L1 and L2, respectively. In chain A, helices H1′, H2′ and H3′ are connected by loops L1′ and L2′ (Fig. 1A). The two chains are structurally very similar to each other, except for the L1 and L1′ regions, with a root mean square deviation (RMSD) of 0.188.

The *Bs*CM_2-CGA structure has several residues in alternate conformations. His73 exists as alternative conformation in both the chains. In chain A, a sulphate ion, contributed by the crystallization buffer, coordinates with ND1 atoms in both conformations. The sulphate ion’s O3 atom interacts with the backbone carbonyl oxygen of His73 and the OE2 side chain atom of Glu77. The O1 atom of sulphate interacts with the NZ atom of Lys76. Altogether, these results indicate that backbone of these residues is rigid and alternative conformation of protein residues allow them to interact with the solvent and the surrounding environment.

#### Dimeric structure

In *Bs*CM_2–CGA, two molecules are present in an asymmetric unit, which form a helical dimeric structure. Previously, few structures of CM_2 dimers from *E. coli* ^24^ and *L. monocytogens* ^9^ have been reported. Those structures are stabilized by cross-helical interactions between two subunits. The dimer interface and assembly analysis performed using PISA server^33^ revealed that almost one third (~2260 Å^2^) of each monomer’s surface area was buried during dimer formation. Dimer assembly has a free energy of assembly dissociation (ΔG^diss^) value of 31.7 kcal/mol, which suggests that the assembly is stable. The solvation free energy gain (ΔG^int^) of the dimer is −37.7 kcal/mol. The negative ΔG^int^ value corresponds to hydrophobic interfaces. The interface interactions are dominated by hydrophobic interactions between monomers. In *Bs*CM_2, residues Leu6, Leu9, Leu16, Ile20, Leu23 and Val30 corresponding to Leu7, Leu10, Leu17, Leu21, Leu24 and Leu31 of *Ec*CM, respectively, participate in these hydrophobic interactions. In previously reported CM_2 dimer structures, helices from both the monomers arrange themselves in an antiparallel manner^9, 24^. Similarly, in *Bs*CM_2, the dimeric interface is formed by antiparallel H1-H1′ and H3-H3′ helix pairs. These interfaces are stabilized by inter-subunit interactions involving hydrogen bonding, salt bridge formation, and hydrophobic interactions. Residues Arg10, Ala13, Asn17, Arg27, Arg50 and Thr70 from chain A form hydrogen bonds with residues Arg10, Asn17, Arg27, Arg50, Met54 and Glu84 of chain B. Residues Asp14 and Arg50 from both the subunits are involved in salt bridge formation.

### Active site and its interaction with inhibitor

In the *Bs*CM_2-CGA structure, two active sites, S1 and S2, are located at the interface of two monomers, as shown in Fig. 1A. Residues from both the monomers contribute in each active site formations. Residues from helices H1, H2, H3, H1′ and loop L1 form the S1 active site, while the S2 active site is formed by residues from the H1′, H2′, H3′, H1 helices and the L1′ loop. The active site contains charged/polar residues, which are highly conserved among all known chorismate mutases. Some hydrophobic residues, proposed to be essential for substrate binding and catalysis, are also present at the active site, but are not conserved. At active site S1, charged/polar residues Arg27, Lys38, Asp47, Arg50, Glu51, and Gln86 from chain B and Arg10 from chain A provide the electrostatic environment for the incoming ligand (substrate/inhibitor). The hydrophobic residues Ile34, Met54, Phe79, Gly82, and Leu83 are also part of the active site S1 (Fig. 1B).

Chlorogenic acid, a structural analogue of the enzyme’s substrate, was co-crystallized with the *Bs*CM_2 protein. Unambiguous electron density of this ligand was observed at both the active sites of the dimer. The ligand interacts with key active site residues through direct or water bridge-mediated hydrogen bonding. As shown in Fig. 1C, at active site S1, the chlorogenic acid makes hydrogen bonds with the side chain of Arg27, Lys38, Gln86, and the main chain atoms of Arg45, Asp47, and Phe79 of chain B. The ligand molecule also interacts with Lys38, Arg50, and Lys80 of chain B, and Arg10 of chain A through water bridge formation. However, at active site S2, along with the above interactions, the ligand forms a direct hydrogen bond with Lys80 and an additional water bridge-mediated hydrogen bond with Gln86 of chain A. In *Bs*CM_2–CIT structure, the active sites of all the chains were occupied by citrate molecule contributed by crystallization buffer. The citrate molecule interacts with the residues Arg27, Lys38, Asp47 and Gln86 of chain A and Arg10 of adjacent chain B through hydrogen bonding (Fig. 1D), and similarly in chain C, and chain D. The citrate molecule also makes additional interactions with the residues Arg27, Arg50 and Phe79 via water bridge formation. It has been previously reported that the citrate inhibits the 90-*Mt*CM protein^34^.

### Comparison with monofunctional *B. subtilis* AroH class of chorismate mutase

Monofunctional *B. subtilis* chorismate mutase (*Bs*AroH) exits as a trimer and forms a pseudo-αβ-barrel core structure via contributions of β-sheets from each monomer^35^. A functional trimer has three active sites formed at the interface of two monomers. However, *Bs*CM_2 forms an entirely helical dimeric structure with two active site clefts which are formed by the residue contributions from both monomers, as previously described.

The *Bs*AroH active site contains charged residues Arg7, Arg90, Arg116, Arg63′ and polar residues Glu78, Tyr108, Lys60′, Thr74′, and Cys75′ (prime (‘) represents the residue from an adjacent chain). Residues Phe57′, Ala59′ and Leu115 are also part of the active site^34^. The *Bs*CM_2 active site consists of similar residues. Polar residues Arg10′, Arg27, Lys38, Asp47, Arg50, Glu51, and Gln86 form the active site along with hydrophobic residues Ile34, Met54, Phe79, and Gly82. The similarity of these active site residues and their chemical nature may allow the binding of similar small molecules to these enzymes.

### Comparison of *Bs*CM_2-CGA with *Ec*CM (catalytically active CM type 2)

In *E. coli*, the N-terminal region of bifunctional P-protein (*Ec*CM) contains a functional ‘Chorismate Mutase II fold’^24^. Although, *Bs*CM_2 shares only 37% sequence identity with *Ec*CM, but they have a similar tertiary structure with an RMSD value of 0.815. Both the proteins’ active sites are formed by the residue contribution from both the monomers. *Bs*CM_2 contains charged residues Arg27, Lys38, Asp47, Arg50, Glu51, and Gln86 from one monomer and Arg10 from the other monomer corresponding to Arg28, Lys39, Asp48, Arg51, Glu52, and Gln88 from one monomer and Arg11 from the other monomer of *Ec*CM. Hydrophobic residues Ile34 (Val35), Met54 (Leu55), Phe79 (Ile81), and Leu83 (Val85) are also present at the active site (residues mentioned in parentheses are corresponding hydrophobic residues present in *Ec*CM). These hydrophobic residues are not conserved between these two proteins. The Ser84 residue of *Ec*CM is also mutated to Gly82 in *Bs*CM_2.

In *Ec*CM, residues Ser15, Asp18, Arg47, Asp48, and Gln88 are key residues that play a supporting role in inhibitor binding. But in *Bs*CM_2, the corresponding residues are Asp14, Asn17, Phe46, Asp47, and Gln86. Variation in these residues may be a contributing factor towards the reduced catalytic activity of *Bs*CM_2. Previous studies of *Ec*CM have shown that mutation of residues Ala32, Val35, Ile81, and Val85 decreases the catalytic activity of the protein^24^. These residues may be important for substrate binding or catalytic activity of the protein. In *Bs*CM_2, the corresponding residues were different, which may result in poor substrate binding/catalysis.

### Comparison of *Bs*CM_2-CGA with *Lm*CM (regulatory CM type 2)

Overall, the folds of *Bs*CM_2 and *Lm*CM are similar with an RMSD of 0.816. In the *Lm*CM domain structure, residues 40–44 of chain A and residues 33–52 of chain B (corresponding to connecting loop L1 of helices H1-H2) were missing, which indicates the structural flexibility of the loop regions. In *Bs*CM_2-CGA, clear density for loop L1 was observed, however, residue 40 was missing from loop L1′. The binding of the substrate analogue may be responsible for stabilizing the loop. Chain A of the *Lm*CM domain was used for further structural analysis. The comparison of the loop region of *Lm*CM with chain A of *Bs*CM_2 showed a small shift in C_α_ atom positions of residues Arg45 (1.0 Å) and Phe46 (0.8 Å), but side chain orientations of these residues were significantly changed. However, chain B of *Bs*CM_2 showed a relatively large shift in both side chain orientations and C_α_ atom positions of residues Arg45 (2.1 Å) and Phe46 (1.2 Å) (values represent C_α_ position shift) in comparison to the corresponding residues of *Lm*CM. It is evident that such a significant shift in the backbone atoms, along with huge variation in side chain orientations in residues of L1 loop, confers plasticity to the enzyme’s active site to accommodate different incoming ligands. The active site residues of both the proteins were similar. As *Lm*CM has been shown to possess a more regulatory role than a catalytic role^9^, the similarities in structure and active site architecture suggest that *Bs*CM_2 may also have a regulatory function in the bifunctional DAHPS enzyme. Previous studies have also reported that *Bs*CM_2 may have a regulatory function^16, 25, 26^.

### Comparison of *Bs*CM_2 with CM domain of *Gsp*DAH7PS

*Bs*CM_2 shares high sequence identity (71%) over 90 amino acids with the CM domain of *Gsp*DAH7PS^25^. Structurally, *Bs*CM_2-CGA align very well with N-terminal 95 residues corresponding to CM domain of *Gsp*DAH7PS with RMSD value of 0.556 Å (5J6F)^25^. The residues demarcating the active site of both structures are highly similar with comparable side chain orientations. In both the structures, ligand (chlorogenic acid in *Bs*CM_2-CGA and prephenate in *Gsp*DAH7PS) occupies the active site with similar network of interactions. Comparison of inter-helical loop regions L1 and L2 of *Bs*CM_2-CGA with *Gsp*DAH7PS showed minimal deviation in their relative orientation with similarly extended helix H1. These observations reinforce that binding of ligand induces the conformational changes with significant rearrangement of secondary structure in *Bs*CM_2. Overall, the structure of *Bs*CM_2-CGA resembles the closed conformation observed in prephenate bound *Gsp*DAH7PS structure.

Interestingly, the structural comparison of chain A of *Bs*CM_2-CIT with CM domain of *Gsp*DAH7PS showed relatively less structural similarity with RMSD value of 0.794 Å (5J6F). The structural variations are primarily confined to inter-helical loop L1 that adopted a less compact extended conformation with shortening of helix H1. This leads to straightening of helix H2 and overall structure resembles the open form of enzyme. Together, these observations clearly indicate that binding of ligand to CM domain induces the significant conformational changes in active site loop L1.

### Molecular dynamics simulation

**Figure 2:**
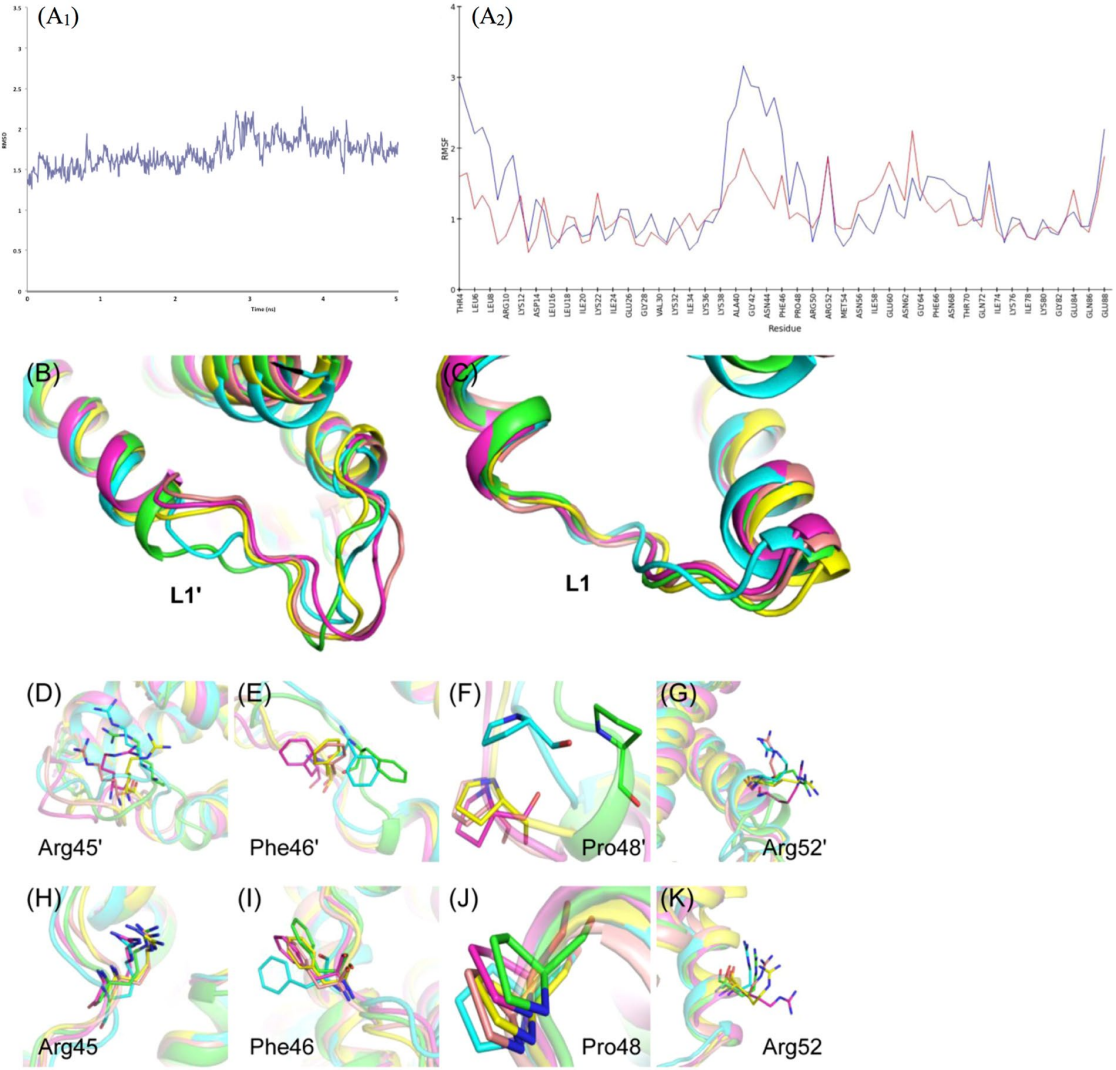
Molecular dynamics simulation results of *Bs*CM_2-CGA. (**A**_1_) RMSD of the backbone atoms with respect to initial structure of the chlorogenic acid bound *Bs*CM_2 structure over 5-ns simulations. (**A**_2_) Plot of RMSF values against individual residues for the conformation generated in production steps with respect to crystal structure. During a simulation backbone shift in loop L1 (**B**) and L1′ (**C**) regions were observed. In chain A, large changes were observed in side chain orientations of Arg45′ (**D**), Phe46′ (**E**), Pro48′ (**F**), and Arg52′ (**G**). However, relatively small changes were observed in chain B (**H–K**). Color code: Green (crystal structure), Cyan (after 1 ns), magenta (after 2 ns), yellow (after 3 ns) and brown (after 4 ns).

As described earlier, chorismate mutase type 2 proteins have an intrinsically disordered loop region, which contributes to active site formation and may be involved in wider substrate specificity and/or regulation. Structural analysis of *Bs*CM_2 revealed a significant shift in the backbone position of L1 and L1′ loop residues, as well as changes in the side chain orientation. To assess the extent of loop movement and side chain flexibility, molecular dynamics (MD) simulation studies of *Bs*CM_2 protein were performed. Discovery Studio suite’s Standard Dynamics Cascade tool was used for the simulation studies using CHARMm forcefield^36^. For solvation, 4327 water molecules were added, along with 21 Na^+^ and 11 Cl^−^ ions to neutralize the system. Conformations generated in a 5 ns production step of simulation were used for analysis. Root mean square deviation (RMSD) of the chlorogenic acid bound *Bs*CM_2 conformations generated during production step of simulation has been found to be fluctuating around an average of 1.9 Å within a narrow range (Fig. 2A_1_). Backbone shift was analyzed by calculation of the root mean square fluctuation (RMSF) for individual residues. A plot of RMSF values for each residue of both the chains is shown in Fig. 2A_2_. Higher RMSF values correspond to higher flexibility of residues. For terminal residues, higher RMSF values were expected because of increased degree of freedom for these residues. From Fig. 2A_2_, it is evident that there is a sudden increase in RMSF values of intrinsically disordered loop residues (Gln41 to Val49). The chlorogenic acid bound at the active site S1 shows a slight deviation in its position. At active site S2, chlorogenic acid maintains its position during the initial 1 ns. In 1ns-2ns time intervals, the caffeic acid moiety shifts towards the loop L1′ and remains intact in the same conformation for the rest of the simulation. This change in the chlorogenic acid conformation may contribute to the shift in the loop L1′ backbone.

In chain A, loop L1′ region residues show significant backbone deviation and side chain orientation change (Fig. 2B). Residues Asn44, Arg45, Phe46 and Asp47 show variations during simulation. During 1 ns-2 ns interval, Asn44 undergoes a large shift in backbone position and also orients its side chain parallel to the active site. Residue Arg45 and Phe46 main chains undergo large shifts away from the active site and also cause significant changes in side chain orientations (Fig. 2D,E). The amino acid Asp47 orients its side chain towards the active site during simulation. Unlike chain A, chain B’s loop L1 region shows only slight deviation in backbone position (Fig. 2C). Arg45 and Phe46 undergo little deviation in side chain orientation, but obtain the initial orientation towards the end of the simulation (Fig. 2H,I). For residue Asp47, main chain fluctuations were observed, but side chain orientation remained intact.

In the crystal structure, Pro48 was positioned in the first turn of helix H2′. During the simulation, helix H2′ undergoes local uncoiling at the first turn (Fig. 2B). Due to uncoiling, Pro48 and Val49 shift their backbone position away from the protein. Large deviations were observed during the initial 2 ns of production step, after which Pro48 maintains a fixed position during the rest of the simulation (Fig. 2F). Towards the end of the simulation, helix H2′ continues to uncoil and increases the size of loop L1′ extended to residue Glu51.

Residue Arg52 of both the chains shows a significant change in side chain orientations during the simulation (Fig. 2G and K). In chain A, Lys76 shows the deviation in side chain orientation during 1 ns-2 ns time intervals, but in chain B, the corresponding residue had no significant change in orientation. *Bs*CM_2 residues Phe46, Val49, Arg52, and Lys76, corresponding to residues Phe46, Leu49, Arg52 and Lys76 of *Lm*CM, show backbone or side chain flexibility. These residues may be involved in domain-domain interface formation between the catalytic and regulatory domains of bifunctional DAHPS.

### Chlorogenic acid’s interaction with monofunctional CM (*Bs*AroH)

**Figure 3:**
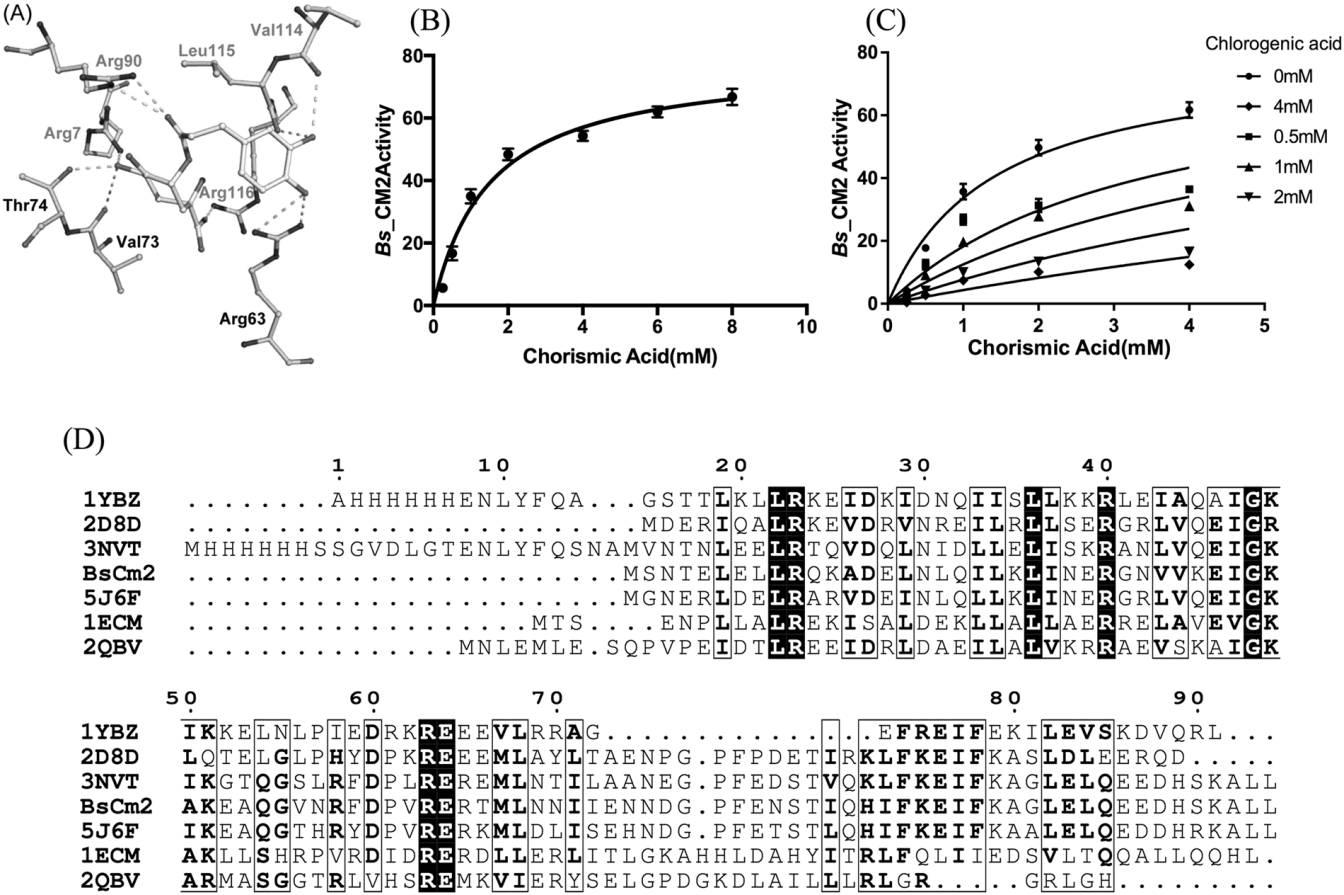
Molecular docking, biochemical assay, and multiple sequence alignment. (**A**) Interaction of chlorogenic acid with monofunctional *Bs*AroH (PDB ID: 2CHT). Chlorogenic acid interacts with residues Arg63, Val73, and Thr74 from one chain (black label) and residues Arg7, Arg90, Val114, Leu115, and Arg116 from the adjacent chain (grey label). (**B**) Michaelis-Menten kinetic curve of *Bs*CM_2. The activity was expressed in terms of International Units (IU)/mg protein. Based on non-linear regression analysis, *k*_*cat*_ and *K*_*m*_ values of *Bs*CM_2 were calculated and found to be 0.78 ± 0.02 S^−1^ and 1514 ± 175 µM, respectively. (**C**) Michaelis-Menten kinetic curve of *Bs*CM_2 in the presence of the inhibitor (chlorogenic acid). Chlorogenic acid concentration was varied from 0.5 mM to 4 mM. The mode of inhibition was found to be competitive with a *K*_*i*_ value 0.33 ± 0.07 mM. (**D**) Multiple sequence alignment of *Bs*CM_2 with *Ec*CM (1ECM), *Lm*CM (3NVT), 90-*Mt*CM (2QBV), *Tt*CM (2D8D), *Gsp*CM (5J6F) and *Pyrococcus furiosus* hypothetical protein (1YBZ).

To assess the plausible interaction of chlorogenic acid with the monofunctional AroH class of CM, the molecule was docked at the active site of *Bs*AroH (PDB ID: 2CHT). Similar to *Bs*CM_2, in *Bs*AroH, the chlorogenic acid’s position is shifted from the transition state analogue (TSA) position. The chlorogenic acid interacts with residues Arg63, Val73, Thr74 from one chain and Arg7, Arg90, Val114, Leu115, and Arg116 from the adjacent chain (Fig. 3A). The similarity of chlorogenic acid’s interaction with both monofunctional *Bs*AroH and *Bs*CM_2 may result in similar binding to both proteins.

### *Bs*CM_2 enzyme assay and activity

The kinetic parameters (*K*_*m*_ and *k*_*cat*_) for *Bs*CM_2 have been calculated and compared with monofunctional *Bs*AroH and chorismate mutase from other reported organisms. The catalytic turnover number, i.e. *k*_*cat*_, was determined to 0.78 ± 0.02 S^−1^ for *Bs*CM_2. *K*_*m*_ was calculated and found to be 1514 ± 175 µM. A plot of Michaelis-Menton kinetics is shown in Fig. 3B. Both *k*_*cat*_ and *K*_*m*_ of *Bs*CM_2 are compared with monofunctional *Bs*AroH. *k*_*cat*_ and *K*_*m*_ values for *Bs*AroH are 46.0 S^−1^ and 67.0 µM, respectively^35^. As expected, the kinetic parameters clearly suggest that *Bs*CM_2 has much lesser activity than *Bs*AroH. The CM activity of *Bs*CM_2 follows Michaelis–Menten kinetics, with a catalytic efficiency (*k*_*cat*_ */K*_*m*_) of 0.5 × 10^3^ M^−1^S^−1^. This catalytic efficiency is three orders of magnitude lower than expected for wild-type CMs^26^. The enzyme activity of *Bs*CM_2 was also assessed in the presence of the substrate analogue, chlorogenic acid. There is a 60% reduction in activity of *Bs*CM_2 after the addition of 1.0 mM chlorogenic acid. Higher concentrations of chlorogenic acid (>4.0 mM) resulted in complete loss of *Bs*CM_2 activity. The kinetic inhibition constant (*K*_*i*_) for chlorogenic acid was 0.33 ± 0.07 mM. A plot of inhibition of *Bs*CM_2 activity by chlorogenic acid is been shown in Fig. 3C. Chlorogenic acid competitively inhibits the *Bs*CM_2 activity with an IC_50_ value 550 µM.

### Isothermal titration calorimetry (ITC)

**Figure 4:**
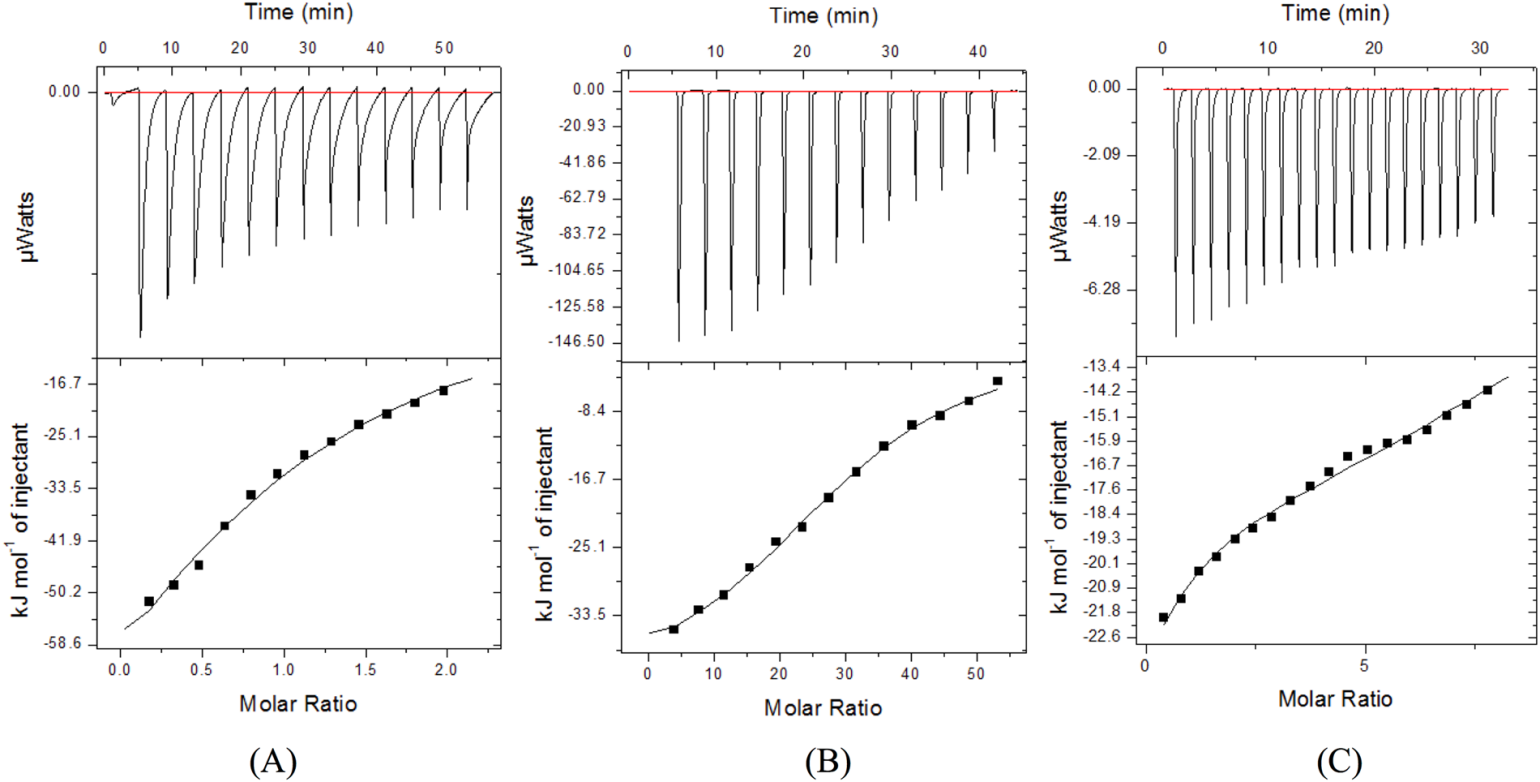
Interaction of *Bs*CM_2 with chorismate, citrate and chlorogenic acid by Isothermal titration calorimetry (ITC). ITC titration data describing the binding of chorismate (**A**), citrate (**B**) and chlorogenic acid (**C**) to *Bs*CM_2. Data is fitted using single binding site model to evaluate dissociation constant (*K*_*D*_). Upper part of each panel shows the thermogram (thermal power Vs time) after baseline correction, while lower part of each panel is binding isotherm (normalized heat Vs molar ratio of reactants).

Binding of *Bs*CM_2 protein with its natural substrate chorismate was performed using ITC. Additionally, binding of *Bs*CM_2 protein with citrate and chlorogenic acid was also assessed. Thermodynamic parameters – enthalpy change (ΔΗ), entropy change (ΔS), Gibbs free energy change (ΔG), and equilibrium dissociation constant (*K*_*D*_) have been determined. Table 1 shows the thermodynamic parameters of binding of ligands. Reactions of these ligands with *Bs*CM_2 are spontaneous in nature as evident by negative ΔG values. Binding of these three compounds with *Bs*CM_2 is driven by enthalpy change (ΔΗ), which indicates that polar interactions are involved during binding. Reactions of these ligands are exothermic in nature as confirmed by the downward trend in ITC profile. The equilibrium dissociation constant for chorismate, citrate, and chlorogenic acid was calculated as 725 µM, 600 µM and 480 µM, respectively. The *K*_*D*_ values showed better affinity of chlorogenic acid for *Bs*CM_2 than citrate and chorismate. Figure 4 shows the thermogram and the fitting curve of chorismate, citrate and chlorogenic acid.

**Table 1:** Binding studies data of *Bs*CM_2 with chorismate, citrate, and chlorogenic acid using ITC.

Compound	Enthlapy change (Joules/mol)	Entropy change (Joules/mol/K)	Free energy (Joules/mol)	Dissociation constant, *K*_*D*_ (µM)
Chorismate	−1.397 × 10^5^	−408	−1.8 × 10^3^	725
Citrate	−4.291 × 10^4^	−82.2	−18.4 × 10^3^	600
Chlorogenic acid	−1.504 × 10^5^	−441	−18.9 × 10^3^	480

### Minimum inhibitory concentration (MIC)

The MIC test for chlorogenic acid was performed with *B. subtilis* strain 3256 using the microdilution method. The MIC value of chlorogenic acid was calculated and found to be 30 ± 5 µg/ml. The moderately high MIC value may be attributed to the low affinity of chlorogenic acid for *Bs*CM_2. It has been reported that chlorogenic acid can inhibit bacterial growth or kill bacterial cells, but the exact mechanism of its inhibitory effect is largely elusive. It has been reported that binding of either the substrate chorismic acid or the product prephenate to the regulatory domain of DAHP synthase of *B. subtilis* regulates the activity of DAHP synthase^29, 30^. As described earlier, the ITC data reveals better binding of chlorogenic acid with *Bs*CM_2 in comparison to chorismic acid, it is possible that chlorogenic acid can inhibit the DAHP synthase enzyme by binding to its regulatory domain, which will further lead to inhibition of the shikimate pathway.

## Discussion

During the course of evolution, nature has developed several mechanisms to regulate metabolic pathways. The molecular mechanisms involved in allosteric regulation of enzyme catalysis have been studied increasingly as more structural information on proteins become apparent. Different classes of DAHP synthases are regulated by different regulation mechanisms^4–9^. In *B. subtilis*, regulation of DAHP synthase is thought to be controlled by its N-terminal domain (*Bs*CM_2), which has residual chorismate mutase activity^26, 27, 29^. Herein, we have reported the crystal structures of the *Bs*CM_2 domain of DAHP synthase from *B. subtilis* in complex with citrate and chlorogenic acid. These crystal structures provide crucial information regarding the interaction of ligands with the protein and subsequent conformational changes, which may be responsible for allosteric regulation of DAHP synthase activity. Additionally, this data paves the way for rational drug design to inhibit the DAHPS activity via targeting the N-terminal chorismate mutase like domain. The active site of *Bs*CM_2 contains polar residues (Arg27, Lys38, Asp47, Arg50, Glu51, and Gln86 from one chain and Arg10 from an adjacent chain), which are highly conserved in all chorismate mutases (Fig. 3D). Additionally, the active site of *Bs*CM_2 also contains some non-conserved hydrophobic residues (Ile34, Met54, Phe79, Gly82, and Leu83) (Fig. 1B). Analysis of *Bs*AroH and *Bs*CM_2 structures revealed that both the structures have different folds, but contain similar active site architecture. Comparison with catalytic and regulatory CM type-2 domains/proteins showed that polar residues at the active site are conserved in all the CM_2 proteins/domains. However, variations were limited to the hydrophobic residues of the active site. These residues may be responsible for differences in catalytic activity in between functional and regulatory domains.

Citrate and the chlorogenic acid both interact with the active site residues through hydrogen bonds and water bridges, as shown in Fig. 1. Position and interactions of the citrate with the protein differ from chlorogenic acid. Chlorogenic acid forms direct hydrogen bonds with Lys38, Arg45, and Lys80, as well as Water Bridge mediated interactions with Lys38 and Lys80. However, citrate only makes a hydrogen bond with Lys38 and does not have any polar contact with Arg45 and Lys80. Additionally, Phe79 backbone atoms form direct interactions with chlorogenic acid, but form water mediated interaction with citrate. These differences in protein-ligand interactions are likely to be responsible for differences in the binding of ligands with protein as observed in ITC studies. According to ITC data, the binding of chlorogenic acid to *Bs*CM_2 is approximately 1.5X stronger than that of chorismic acid, and 1.25X stronger than that of citrate molecules.

The comparison of catalytic efficiency of *Bs*CM_2 with previously reported parameters of monofunctional *Bs*AroH^37^ suggests that the *Bs*CM_2 enzyme has three fold less catalytic efficiency than *Bs*AroH. These findings suggest that *Bs*CM_2 may have regulatory role as fusion partner with *Bs*DAHPS^26, 27, 29^. As per earlier reported data, chlorogenic acid is an antibacterial compound, but the mechanism of its inhibitory action is poorly understood^32^. The activity of *Bs*CM_2 enzyme was assessed in the presence of chlorogenic acid, and it was found that chlorogenic acid inhibits the *Bs*CM_2 enzyme competitively with a *K*_*i*_ of 0.33 ± 0.07 mM.

As both *Bs*CM_2 and *Bs*AroH share similar active site architecture, it is plausible that chlorogenic acid might also bind to the *Bs*AroH enzyme and inhibit its function. Docking studies to assess the interactions of chlorogenic acid with *Bs*AroH showed that chlorogenic acid interacts with the active site residues of the protein in a manner similar to substrate. The MIC of chlorogenic acid for a *B. subtilis* strain was found to be 30 ± 5 µg/ml. These results suggest that chlorogenic acid and its structural analogues may be developed as potential drug molecules.

### Helices H1-H2 connecting Loop L1 flexibility

**Figure 5:**
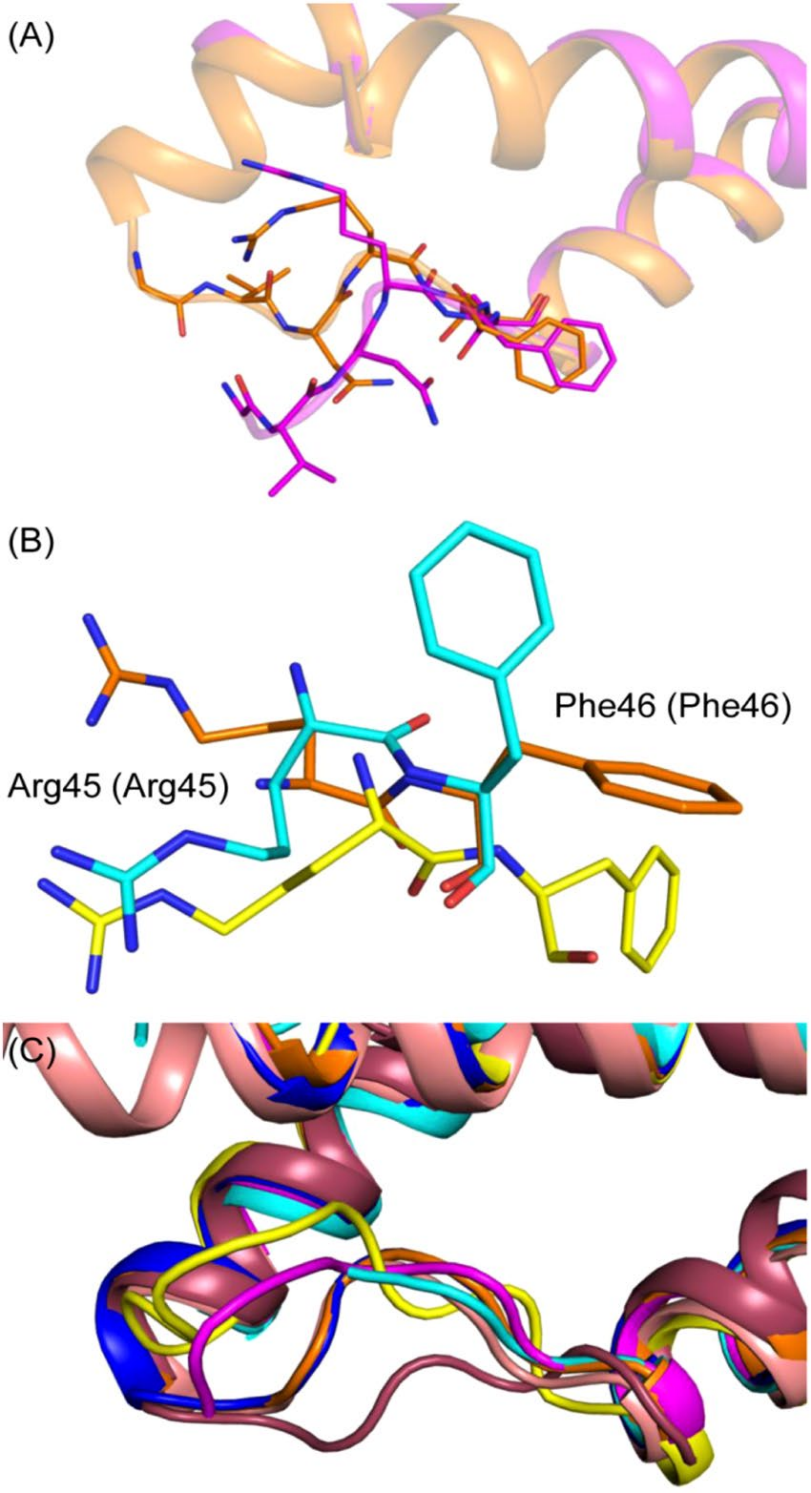
Flexibility of loop L1. In panel (A), superimposition of two chains of the *Bs*CM_2-CGA structure shows the deviation in backbone position of residues corresponding to loop L1. Panel (B) depicts the conformational flexibility of loop L1 residues Arg45 and Phe46 in different structures (parentheses are corresponding residues in *Lm*CM domain). Superimposition of *Bs*CM_2-CGA chainB (orange), *Bs*CM_2-CIT chainA (yellow) and *Lm*CM domain (PDB ID: 3NVT) chainA (cyan) reveals the flipping of side chains of residues. In panel (C), superimposition of CM_2 domains has shown that loop L1 exists in different conformations. This backbone deviation and side chain flipping may participate in regulation of DAHPS activity by CM_2 domains. *Bs*CM_2-CGA chainA (magenta), *Bs*CM_2-CIT chainB (blue), *E. coli* P-protein’s CM domain (PDB ID: 1ECM) chainA (salmon) and *Mt*CM (PDB ID: 2VKL) (raspberry) were used for structural analysis to assess the conformational flexibility of loop L1.

In previous studies, the *Lm*CM_2 domain structure had H1-H2 connecting loop regions disordered in both the chains, which emphasizes the flexible nature of the this loop. In *Bs*CM_2-CGA, chlorogenic acid interacts with active site loop L1 residues Arg45 and Asp47 directly and via water bridges mediated by hydrogen bonds. This extensive hydrogen-bonding network might be stabilizing loop L1. In two chains of *Bs*CM_2-CGA structures, the position of loop region residues varies significantly relative to each other (Fig. 5A). Between chain A and chain B, corresponding C_α_ atom positions of residues Gly42, Val33, Asn44, Arg45, and Phe46 deviated by 6.8, 8.9, 4.6, 1.5 and 1.1 Å, respectively. Structural comparison with other proteins with the same fold also showed the deviation in the backbone position of loop region residues (Fig. 5B,C). The huge shift in backbone position suggests the plasticity of active site loop L1.

Molecular dynamics simulations of the *Bs*CM_2-CGA structure showed deviations in backbone position and side chain orientations of loop region residues. In the *Bs*CM_2-CGA structure, loop L1′ has a higher B-factor than loop L1. During simulation, large deviations were observed in the loop L1′ region, but loop L1 showed only slight deviations in backbone atoms (Fig. 2B,C). In chain A, helix H2′ undergoes uncoiling at the first turn, thus increasing the size of loop region. Significant backbone movement was observed for the longer loop. This long loop region contains residues Phe46, Val49 and Arg52, which correspond to the Phe46, Leu49, and Arg52 of *Lm*CM. These residues may be involved in domain-domain interface formation along with Lys76. The residues of loop L1′ contribute to the active site formation, and flexibility of these residues provide plasticity to the active site and may play an important role in the wider substrate specificity.

### Regulation mechanism

Previously, Light *et al*. described plausible regulatory mechanisms of DAHPS activity by the CM_2-like domain^9^. They proposed two hypotheses by which the CM_2-like domain may regulate DAHPS activity. First, domain linker mediated direct inhibitor conformation change transmission and second, domain-domain interface mediated allosteric regulation. They have also summarized the arguments against and in support of both the mechanisms.

Recently, Nazmi *et al*. provided functional and structural evidences suggesting that CM mediated regulation of DAHPS activity results from the significant inter-domain conformational changes upon binding of ligand to the CM domain^25^. These changes make the DAHPS active site inaccessible to substrate, therefore inhibiting the DAHPS activity.

**Figure 6:**
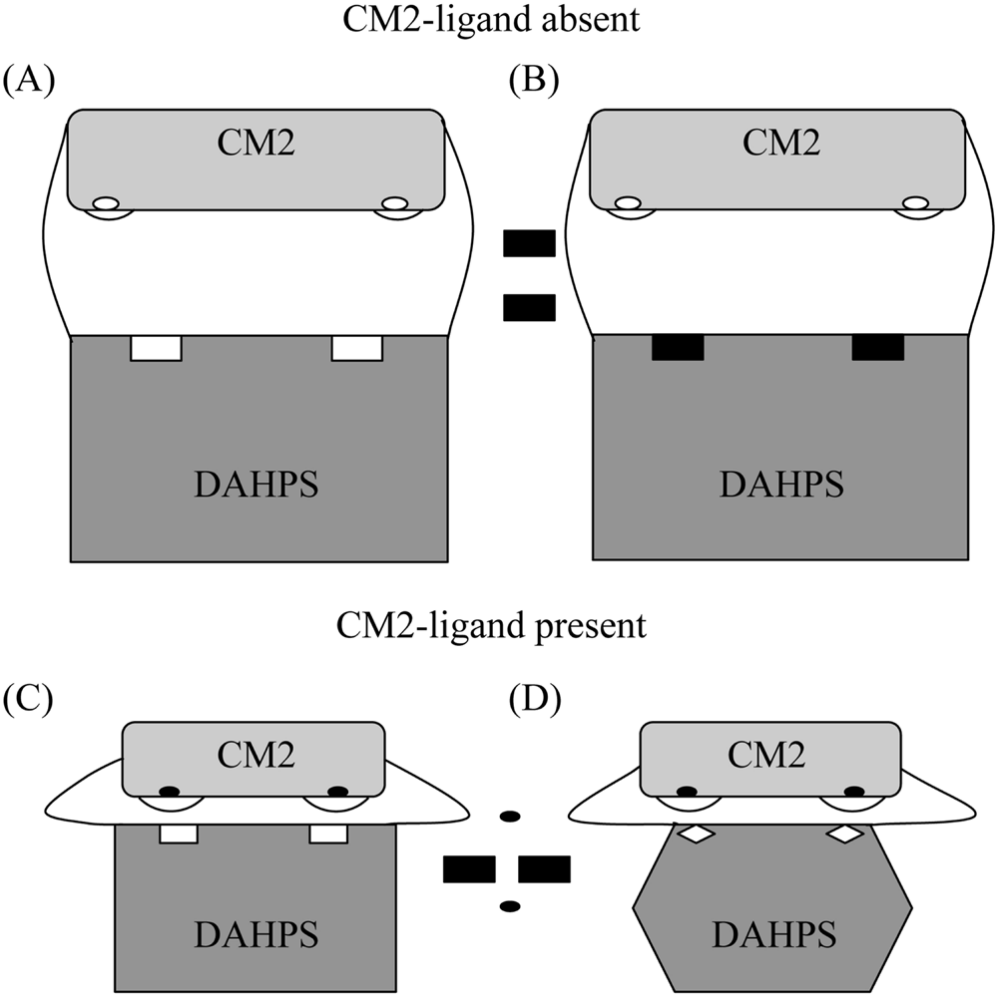
Regulation of DAHPS enzyme activity by the CM2 domain. Panel (A) shows the basic architecture of DAHPS-CM domain dimers in the absence of a CM2-ligand. Panel (B) shows the substrate (rectangular black box) binding at the active site of the DAHPS domain in the absence of a CM2-ligand. In panel (C), CM2-ligand (oval black box) binds at the active site of CM2, which leads to the conformational changes in linker region and loop L1, which connects helices H1-H2. The linker region adopts the kinked conformation and brings the DAHPS and CM2 domains near to each other. Loop L1 extends outward and flips side chains of loop residues. Loop L1 mediates the domain-domain interface formation between DAHPS and CM2 domains and blocks the DAHPS-substrate access to the DAHPS-active site. As shown in panel (D), the DAHPS domain undergoes conformational changes, which will hinder in the binding of substrate at the active site of this domain. For simplicity, the only dimer of DAHPS has been shown instead of the tetramer.

From our structural and molecular dynamics simulation studies, we reinforce the hypothesis of the domain-domain interface interaction mediated DAHP synthase regulation mechanism in *B. subtilis*. Structural and molecular dynamics simulation studies revealed the flexibility of loop connecting helices H1 and H2. Structural analysis of *Lm*DAHPS and *Gsp*DAH7PS showed that a linker region connecting the two domains is highly flexible and can exist in both open and close forms^9, 25^. On the basis of our crystal structures, we propose that in the absence of ligand binding in the *Bs*CM_2 active site, the inter-helical loop L1 exist in an extended form leads to the shortening of helix H1 and the linker adopts an open conformation. Upon binding of the ligand at the *Bs*CM_2 active site, the loop L1 becomes shorter and acquires a more compact form, result in longer helix H1. The change in the orientation of loop L1 leads to rearrangement of side chains of loop region residues, and linker regions adopt a closed conformation, bringing the two domains near to each other. The domain-domain interface formation may limit the access of the substrate to the DAHPS active site, inhibiting its activity. The ligands bound at the active site of *Bs*CM_2 can be its substrate, product or their analogues. Binding of any of these ligands may allow *Bs*CM_2 to form a domain-domain interface with the DAHPS domain and limit DAHPS catalytic activity (Fig. 6).

### Importance

Previously, it has also been reported that the AroH and the AroQ type chorismate mutase enzymes share the functional uniformity and similar affinity towards common substrate. Therefore, inhibitors simultaneously targeting both types of CM enzymes can be developed. Targeting two different enzymes of the same shikimate pathway with a common inhibitor may limit the availability of essential aromatic amino acids and metabolites required by pathogenic microbes, thus causing death. Also, targeting multiple enzymes using a single inhibitor/drug reduces the possibility of developing drug resistance. The crystal structures of *Bs*CM_2 complexed with chlorogenic acid, and citrate, provide insight into the mode of protein-ligand interactions. This information can be utilized for designing analogues of these ligands for potent *Bs*CM_2 inhibitor development.

## Materials and Methods

*Bacillus subtilis s*train 3256 was purchased from Microbial Type Culture Collection and Gene Bank (MTCC No.1427). All nucleic acid manipulations were performed using standard procedures^38^. All enzymes (DNA polymerase, restriction enzymes, T_4_ DNA ligase, *etc*.) were purchased from New England BioLabs. Qiagen kits were used for plasmid isolation, purification, and gel extraction. *Escherichia coli* DH5α cells and *E. coli* BL21 (DE3) cells were obtained from Novagene. Primer procurement and DNA sequencing was from Eurofins (India). All other chemicals and reagents were purchased from Sigma.

### Construction of plasmid

306 bp long DNA fragment encoding the AroQ class of CM, type II like N-terminal domain of BsDAHP synthase was amplified by standard PCR procedures using *B. subtilis* strain 3256 genomic DNA as a template, with forward (P1) and reverse (P2) primers containing NdeI and XhoI sites (underlined), respectively (P1-CAT TCT CAT ATG AGC AAC ACA GAG TTA; P2- CAT TCT CTC GAG TTA GTG ATC TTC TTC CTG). Amplified product was purified and digested with NdeI and XhoI restriction enzymes. Digested product was extracted from the gel using a Qiagen gel extraction kit. Digested product was ligated to similarly digested *pET*28c plasmid. The ligation product was transformed into *E*. *coli* DH5α cells. The presence of the *Bs*CM_2 gene in the plasmid was confirmed by restriction digestion and the gene sequence was verified by DNA sequencing. The plasmid with the correct sequence, pET28c/*Bs*CM_2, was used to transform chemically competent *E. coli* BL21 (DE3) cells.

### Protein expression and purification

*E. coli* BL21(DE3) cells with the desired plasmid (pET28c/*Bs*CM_2) were grown in kanamycin (50 µg/ml) containing LB (Luria–Bertani) medium at 37 °C. When OD_600_ of the culture reached to ~0.8, cells were induced with 0.4 mM isopropyl-β-D-thiogalactoside and kept for growth at 37 °C for 4 h. Cells were harvested and stored at −80 °C. Cell pellets were suspended in lysis buffer (buffer A) composed of 25 mM Tris buffer (pH 7.5), 200 mM NaCl and 10 mM imidiazole (pH 7.5). Cell pellets were disrupted using high-pressure cell disrupter (Constant Systems Limited, UK), with 20 kPSI pressure in the minicell for 60 s, followed by centrifugation to remove cell debris (20817 × g, 50 min, 4 °C). The clarified supernatant was loaded onto a 5 ml HisTrap Ni-NTA agarose affinity column (GE Healthcare) pre-equilibrated with buffer A, and elution was performed with a linear or step gradient of 50–250 mM imidazole in buffer A. The fractions containing the desired protein, as confirmed by SDS-PAGE, were pooled and dialysed overnight against 2 litres of 25 mM Tris buffer (pH 7.5). The protein was further concentrated up to approximately 5 mg/ml using a 3 kDa cutoff Amicon Ultra-15 concentrator (Millipore, Bedford, Massachusetts, USA). The concentrated protein sample was loaded onto a HiLoad 16/60 prep grade Superdex 75 size-exclusion chromatography column (GE Healthcare), pre-equilibrated with 25 mM Tris-HCl (pH 7.5) and 50 mM NaCl, operated by UNICORN software. The absorbance of eluted protein samples was continuously monitored spectrophotometrically at 280 nm. The purity of the major chromatogram peak collected in different fractions was further analyzed with 15% SDS-PAGE. The purified protein was concentrated up to 18 mg/ml for crystallization trials.

### Crystallization and data collection

For crystallization, a sitting drop vapor diffusion method was used in which each well was loaded with 1.0 μl of protein sample and an equal volume of the reservoir solution, equilibrated against 50 μl reservoir solutions. The initial screening for the crystallization of *Bs*CM_2 was carried out using various commercial kits from Hampton Research (Hampton Research Inc., Aliso Viejo, CA). Crystal hits were observed in conditions containing 1 M ammonium sulphate, 0.1 M potassium sodium tartrate, and 0.1 M sodium citrate pH 5.8 as the buffer at 20 °C in 15 days. In order to make a complex with the substrate analogue, *Bs*CM_2 was co-crystallized with chlorogenic acid (25 mM). *Bs*CM_2 protein mixed with chlorogenic acid at a 1:1 molar ratio was incubated at 4 °C for 2–4 hrs prior to crystallization.

Prior to data collection, crystals of *Bs*CM_2-citrate and *Bs*CM_2-chlorogenic acid were cryoprotected by brief soaking with well solutions containing 20% glycerol and 10% ethylene glycol, respectively. X-ray diffraction was performed at the home source, with a Bruker-Nonius Microstar H rotating-anode X-ray generator (CuK_α_ = 1.54 Å), at 100 K temperature. Diffraction data from crystals were collected using a MAR345dtb image plate detector. Diffraction data were processed using the HKL2000 program^39^.

### Structure solution and refinement

Protein structures were solved and refined using PHENIX^40^ and CCP4i^41^ suites. MOLREP^42^ was used for molecular replacement; and phenix.refine^39^ and REFMAC5^43^ programs were used for refinement. COOT was used for visualization of electron density maps and manual model building^44^. The *Bs*CM_2 domain shares 64% identity with N-terminal CM domain of bifunctional DAHPS from *Listeria monocytogens* (PDB ID: 3NVT)^9^. The *Lm*CM domain structure (modified 3NVT containing only residues 25–108) was used as template for initial phase determination using molecular replacement. The *Bs*CM_2-chlorogenic acid complex (*Bs*CM_2-CGA) structure was refined up to R_work_ and R_free_ values of 17.73% and 21.52%, respectively. Similarly, the *Bs*CM_2 (*Bs*CM_2-CIT) structure was refined upto R_work_ and R_free_ values of 18.32% and 23.32%, respectively. Data collection, structure refinement, and validation statistics of both structures are shown in Table 2. For structural validation, stereochemical properties of crystal structures were accessed through MolProbity^45^. PISA (Protein Interfaces, Surfaces and Assemblies) tool was used for dimer interface interactions analysis^46^. The PyMOL visualization tool was used for structural analysis and figure preparation [Schrödinger, LLC]^47^.

**Table 2:** Summary of data collection, structure refinement, and validation statistics.

	*Bs*CM_2-CGA	*Bs*CM_2-CIT
PDB ID	5GMU	5GO2
**Crystallographic data**		
Space group	*P*2_1_	*P*1
Resolution	1.80	1.90
**Cell dimensions**		
*a, b, c* (Å)	35.7, 47.0, 56.8	45.1, 45.4, 48.3
*α, β*, *γ* (°)	90, 107.23, 90	81.65, 82.87, 78.24
Unique reflections (Last shell)	16372 (716)	26625 (2394)
Completeness (%) (Last shell)	98 (88)	93 (81)
*R*_meas_^a^	0.07	0.08
*R*_pim_^b^	0.04	0.05
I/σ (Last shell)	37.2 (3.76)	19.80 (2.4)
Multiplicity (Last shell)	3.4 (2.7)	3.1 (2.1)
**Refinement**		
No. of Residues	171	348
Water molecules	116	123
Resolution range (Å)	50.00–1.80 (1.83–1.80)	24.66–1.90 (1.97–1.90)
*R*_work_ (%)	17.73	18.32
*R*_free_ (%)	21.52	23.32
RMSD on bond lengths (Å)	0.016	0.007
RMSD on bond angles (Å)	1.769	0.760
**Average** ***B*** **-factors (Å** ^**2**^ **)**		
ChainA	20.64	44.40
ChainB	19.32	37.75
ChainC	—	45.49
ChainD	—	43.00
Water atoms	40.91	48.45
**Ramachandran plot (%)**		
Favored	100	99.71
Outliers	0	0.29

*Statistics for the highest-resolution shell are shown in parentheses.

^a^ *R*_*meas*_ = ∑_*hkl*_{N/(N − 1)}^½^∑_*i*_|I_*i,hkl*_− < I_*hkl*_ > |/∑_*hkl*_∑_*i*_ |I_*i,hkl*_|.

^b^ *R*_*pim*_ = ∑_*hkl*_[1/{N_*hkl*_ − 1}]^½^∑_*i*_ |I_*i,hkl*_− < I_*hkl*_ > |/∑_*hkl*_∑_*i*_|I_*i,hkl*_|.

### Molecular dynamics simulation

To study the backbone flexibility and side chain orientation of the loop connecting helices H1-H2, the *Bs_*CM2-CGA protein structure in the chlorogenic acid bound form was simulated using Discovery Studio (Accelrys Inc., San Diago, CA, USA) suite’s simulation protocol. Before the simulation, the protein structure was prepared by adding hydrogen atoms and was typed with CHARMm forcefield^36^. Typed protein was solvated with the explicit periodic boundary solvation model in an orthorhombic cell whose edges had minimum 7 Å distance from protein surface. Sodium (Na^+^) and chloride ions (Cl^−^) were also added to the system as counter ions for neutralizing the system. Solvated protein was subjected to the molecular dynamics simulation using Standard Dynamics Cascade protocol. The protocol consists of two minimization steps along with heating, equilibrium, and production steps. Minimazation1 and minimization2 steps were performed using the steepest descent algorithm for 1000 cycles and Powell algorithm for 2000 cycles, respectively. The system was heated for 120 ps from 50 K to 300 K temperature. Subsequently, the system was equilibrated for 100 ps at 300 K temperature under constant pressure conditions. Finally, 5 ns production step was performed on isobaric-isothermal ensemble (NPT) at 300 K temperature. The results were saved at every 5 ps time interval. For the entire simulation, 2 fs time step was used. The Particle Mesh Ewald (PME) method was used for handling electrostatic interactions with non-bond list radius of 14 Å. SHAKE constraints were used for constraining hydrogen atoms involving bonds.

### Molecular docking

To assess the plausible interactions of chlorogenic acid with the active site of monofunctional *B. subtilis* AroH class of chorismate mutase (*Bs*AroH), molecular docking was performed using AutoDock4^48^. The crystal structure of *Bs*AroH (PDB ID: 2CHT) was used for the same^35^. For docking, protein and ligand were prepared using AutoDock Tools-1.5.6. A grid centered at (51.787, 25.245, 45.348) with dimensions 40X40X40 (Å^3^) and 0.375 Å spacing was used for map calculations. Lamarchian Genetic Algorithm was used for docking. AutoGrid4 and AutoDock4 programs were executed for calculating energy maps and docking of ligands, respectively. Analysis of docking conformations was performed by converting docking conformations into *Pdbqt* files and visualizing in PyMol.

### Enzyme assay

Chorismate mutase activity was measured by using the fixed-point assay, with minor modifications^49^. The standard assay was carried out in assay buffer (50 mM Tris–HCl, pH7.5) supplemented with 2 mM chorismic acid in a final volume of 200 μl. The mixture was then incubated with shaking at 37 °C for 5 min, and enzyme was further added to initiate the reaction. The reaction was terminated by the addition of 0.2 ml of 1 M HCl. The reaction mixture was left over at 37 °C for 15 min, and finally terminated by the addition of 0.5 ml of 1 M NaOH. The absorbance of samples were recorded at 320 nm using Agilent Carry 300 UV/Vis Spectrophotometer, to monitor the formation of phenylpyruvate. For calculations, 17500 M^−1^.cm^−1^ was used as the extinction coefficient at 320 nm (ε320 nm) for phenylpyruvate^49^. Blank readings were taken without the addition of enzyme to account non-enzymatic conversion of the chorismate to the prephenate.

### Isothermal titration calorimetry

ITC experiments were performed on MicroCal ITC 200 unit (GE Healthcare) operating at 298 K temperature. The binding of a *Bs*CM_2 protein with its natural substrate chorismic acid, and substrate analogue chlorogenic acid were studied. Protein sample was dialyzed against Buffer I (25 mM Tris HCl, pH 7.5) and concentrated up to 8 mg/ml concentration (750 μM). All solutions were degassed before use. The reference cell was filled with buffer I. The Titration reactions were performed using *Bs*CM_2 protein in ITC sample cell and ligands (Chorismate: 7.5 mM, Citrate: 10 mM and Chlorogenic acid: 2.5 mM) in a syringe with 200 rpm stirring speed and initial delay of 60 s. A total of 15–20 injections were made with first one of 0.5 μl and the rest of 2.0 μl each. The first injection was treated as scouting injection to calibrate the iTC200 instrument. The time interval between each injection was 150 s, and the reference cell power was set to 8 μW. ITC data were analyzed using single binding site model using Origin 7.0 software.

### Minimum inhibitory concentration (MIC)

An MIC test for the chlorogenic acid on *B. subtilis* was performed using the Broth micro dilution method on a 96 well microtiter plate^50^. A concentration gradient (10 µg/ml – 200 µg/ml) of chlorogenic acid was tested. A single colony of *B. subtilis* strain 3256 was used to inoculate the nutrient broth medium. Correlation between OD_600_ and microbial number was determined using serial dilution. The OD_600_ of overnight grown culture was adjusted to 1 by dilution with PBS (1x) buffer. The culture was then serially diluted from 10^−1^ to 10^−7^ and 100 µl from the last four dilutions (10^−4^ to 10^−7^) were plated on nutrient agar plates followed by overnight incubation at 37 °C. Colonies were counted and colony-forming-units (cfu) per ml of culture were calculated for each dilution. Correlation between OD_600_ and cfu per ml was then calculated. Bacterial suspensions having 1 × 10^8^ cfu per ml were used to inoculate 96 well microtiter plates. Chlorogenic acid was dissolved in PBS buffer (1x). 100 µl of broth solution was added to all wells. 50 µl of inhibitor solution was used in each well, except growth control (broth and inoculum) and sterility control (broth only) wells. 50 µl of bacterial suspension adjusted to 1 × 10^6^ cfu per ml was used, giving 5 × 10^5^ cfu per ml in the final inoculum. Microtiter plates were then incubated at 37 °C for 16–20 h.
